# Impact of obesity on procedural safety and 1-year outcomes after pulsed-field ablation for atrial fibrillation

**DOI:** 10.1093/europace/euag288

**Published:** 2026-10-29

**Authors:** Grégoire Massoullié, Corentin Chaumont, Mikael Laredo, Bruno Pereira, Olivier Thomas, Philippe Maury, Pascal Defaye, Serge Boveda, Eloi Marijon, William Escande, Antoine Roux, Franck Raczka, Yves Guyomar, Pierre Jaïs, Pierre Ollitrault, Mathieu Granier, Samir Fareh, Hugo Marchand, Bertrand Pierre, Selim Abbey, Antoine Noel, Antoine Da Costa, Fabrice Extramiana, Olivier Cesari, Laure Champ-Rigot, Ziad Khoueiry, Thibault Villemin, Sarah Traullé, Frédéric Treguer, Hugues Bader, Jean-Claude Deharo, Bruno Degand, Benoît Guy-Moyat, Didier Klug, Estelle Gandjbakhch, Alexandre Zhao, Maxime Beneyto, Sandrine Venier, Stéphane Combes, Antoine Lepillier, Marc Mielczarek, Nicolas Clementy, Aymeric Menet, Frédéric Sacher, Christian De Chillou, Romain Eschalier, Frédéric Anselme

**Affiliations:** Cardiology Department, CHU Clermont-Ferrand, 58 rue Montalembert, Clermont-Ferrand F-63000, France; Institut Pascal, Université Clermont Auvergne, CNRS, SIGMA Clermont, 4 avenue Blaise Pascal, TSA 60026 / CS 60026, Aubière Cedex F-63178, France; Department of Cardiology, Rouen University Hospital and UNIROUEN, INSERM U1096, Rouen, France; Institut de Cardiologie, Hôpital Pitié-Salpêtrière, AP-HP Sorbonne Université, Paris, France; Department of Biostatistics, CHU Clermont-Ferrand, Clermont-Ferrand F-63000, France; Department of Cardiology, GHP Ambroise Paré Hartmann, Neuilly-sur-Seine, France; Department of Cardiology, Toulouse University Hospital, Toulouse, France; Cardiology Department, Grenoble-Alpes University, University Hospital, Grenoble, France; Heart Rhythm Management Department, Clinique Pasteur, Toulouse, France; Division of Cardiology, European Georges Pompidou Hospital, Paris, France; Department of Cardiology, Centre Cardiologique du Nord, Saint-Denis, France; Department of Cardiology, Pôle Santé République, Clermont-Ferrand, France; Department of Cardiology, Clinique du Millénaire, Montpellier, France; Cardiology Department, Datacoeur Research Team, ETHICS Laboratory, GHICL Hôpital Saint Philibert, Lille, France; Cardiac Arrhythmia Department, IHU Liryc, Univ. Bordeaux, INSERM 1045, CHU de Bordeaux, Bordeaux, France; Department of Cardiology, Caen University Hospital, Caen, France; Department of Cardiology, CHU de Montpellier, Montpellier, France; Department of Cardiology, Hôpital de la Croix-Rousse, Lyon, France; Department of Cardiology, Institut Arnault Tzanck, Saint-Laurent-du-Var, France; Department of Cardiology, CHU de Tours, Tours, France; Department of Cardiology, Hôpital Privé du Confluent, Nantes, France; Department of Cardiology, Polyclinique Saint-Laurent, Rennes, France; Department of Cardiology, CHU de Saint-Étienne, Saint-Étienne, France; Department of Cardiology, AP-HP Hôpital Bichat, Paris, France; Department of Cardiology, Clinique Saint Augustin, Bordeaux, France; Department of Cardiology, Caen University Hospital, Caen, France; Department of Cardiology, Clinique Saint-Pierre, Perpignan, France; Department of Cardiology, Polyclinique Reims-Bezannes, Bezannes, France; Department of Cardiology, Clinique Victor Pauchet, Amiens, France; Department of Cardiology, Clinique Saint-Joseph, Trélazé, France; Department of Cardiology, Centre Hospitalier de Pau, Pau, France; Department of Cardiology, CHU de Marseille, Marseille, France; Department of Cardiology, CHU de Poitiers, Poitiers, France; Department of Cardiology, CHU de Limoges, Limoges, France; Department of Cardiology, CHU de Lille, Lille, France; Institut de Cardiologie, Hôpital Pitié-Salpêtrière, AP-HP Sorbonne Université, Paris, France; Department of Cardiology, GHP Ambroise Paré Hartmann, Neuilly-sur-Seine, France; Department of Cardiology, Toulouse University Hospital, Toulouse, France; Cardiology Department, Grenoble-Alpes University, University Hospital, Grenoble, France; Heart Rhythm Management Department, Clinique Pasteur, Toulouse, France; Department of Cardiology, Centre Cardiologique du Nord, Saint-Denis, France; Department of Cardiology, Pôle Santé République, Clermont-Ferrand, France; Department of Cardiology, Clinique du Millénaire, Montpellier, France; Cardiology Department, Datacoeur Research Team, ETHICS Laboratory, GHICL Hôpital Saint Philibert, Lille, France; Cardiac Arrhythmia Department, IHU Liryc, Univ. Bordeaux, INSERM 1045, CHU de Bordeaux, Bordeaux, France; Department of Cardiology, University Hospital Nancy, Nancy, France; Cardiology Department, CHU Clermont-Ferrand, 58 rue Montalembert, Clermont-Ferrand F-63000, France; Institut Pascal, Université Clermont Auvergne, CNRS, SIGMA Clermont, 4 avenue Blaise Pascal, TSA 60026 / CS 60026, Aubière Cedex F-63178, France; Department of Cardiology, Rouen University Hospital and UNIROUEN, INSERM U1096, Rouen, France

**Keywords:** Atrial fibrillation, Pulsed-field ablation, Obesity, Body mass index, Catheter ablation, Arrhythmia recurrence

## Abstract

**Aims:**

Obesity is associated with atrial fibrillation (AF), atrial remodelling, and cardiometabolic comorbidities. Whether baseline body mass index (BMI) influences procedural safety and effectiveness of pulsed-field ablation (PFA) remains uncertain. We evaluated the association of baseline BMI with procedural characteristics, periprocedural safety, and 1-year arrhythmia recurrence after first-time PFA for AF.

**Methods and results:**

FRANCE-PFA is a nationwide registry including consecutive patients undergoing first-time AF ablation with a pentaspline PFA catheter. Patients with available BMI were categorized as <25, 25 to <30, 30 to <35, or ≥35 kg/m^2^. Periprocedural complications and 1-year atrial arrhythmia recurrence were assessed across BMI categories. Among 5223 patients, BMI was available in 4573 (87.6%). Higher BMI was associated with greater cardiometabolic burden, more persistent AF, larger atria, and more extensive ablation strategies, including fewer pulmonary vein isolation-only procedures and more additional lesion sets (all *P* < 0.001). Periprocedural complications remained infrequent (1.0%), with no significant difference across BMI categories. At 1 year, recurrence was driven mainly by AF type rather than obesity. Within paroxysmal and non-paroxysmal AF, recurrence rates were comparable between patients with BMI < 35 and ≥35 kg/m^2^ (13.8% vs. 9.1% and 22.9% vs. 23.3%, respectively; log-rank *P* = 0.925). Severe obesity was not associated with recurrence after multivariable adjustment (HR, 0.86; 95% CI, 0.65–1.14; *P* = 0.299), with similar findings when BMI was analysed continuously.

**Conclusion:**

In this nationwide PFA cohort, higher BMI identified a more advanced AF phenotype and more extensive ablation strategies but was not independently associated with periprocedural complications or 1-year arrhythmia recurrence.

## Graphical Abstract

**Graphical Abstract:**
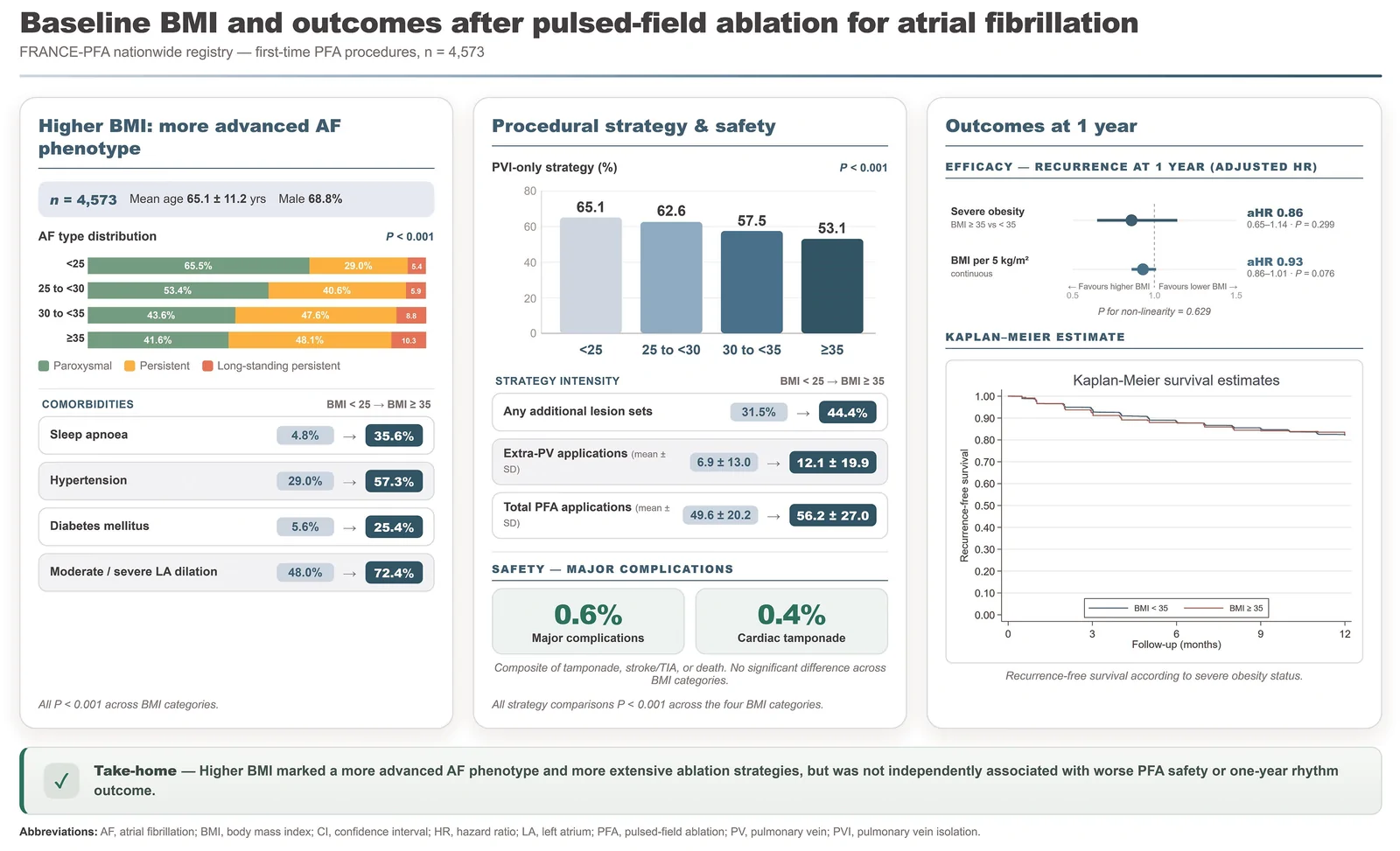
Baseline body mass index (BMI) and 1-year outcomes after first-time pulsed-field ablation (PFA) for atrial fibrillation (AF) in the FRANCE-PFA registry. Among 4573 patients with available baseline BMI (87.6% of 5223 screened; mean BMI 27.6 ± 5.1 kg/m^2^), outcomes were analysed across World Health Organization BMI categories (<25, 25 to <30, 30 to <35, and ≥35 kg/m^2^) and according to severe obesity (BMI ≥ 35 kg/m^2^). (Left) Higher BMI was associated with a more advanced AF phenotype: the proportion of persistent AF and the prevalence of sleep apnoea, hypertension, diabetes mellitus, and moderate-to-severe left atrial (LA) dilation increased progressively (all *P* < 0.001). Values compare BMI < 25 vs. BMI ≥ 35 kg/m^2^. (Middle) Higher BMI was associated with more extensive ablation: PVI-only strategy decreased from 65.1 to 53.1%, whereas additional lesion sets (31.5–44.4%), extra-pulmonary vein applications (6.9–12.1), and total PFA applications (49.6–56.2) increased (all *P* < 0.001). Major complications (cardiac tamponade, stroke/transient ischaemic attack, or death) occurred in 0.6%, with no significant difference across BMI categories (*P* = 0.539). (Right) Severe obesity was not independently associated with 1-year arrhythmia recurrence: aHR 0.86 (95% CI, 0.65–1.14; *P* = 0.299) for BMI ≥ 35 vs. <35 kg/m^2^ and 0.93 (95% CI, 0.86–1.01; *P* = 0.076) per 5 kg/m^2^ increase. No non-linear association (*P* for non-linearity = 0.629); Kaplan–Meier recurrence-free survival did not differ by severe obesity status (log-rank *P* = 0.925). Cox models adjusted for age, sex, sleep apnoea, hypertension, diabetes mellitus, prior stroke/TIA, vascular disease, structural heart disease, and AF type. Abbreviations: aHR, adjusted hazard ratio; AF, atrial fibrillation; BMI, body mass index; CI, confidence interval; HR, hazard ratio; LA, left atrium; PFA, pulsed-field ablation; PV, pulmonary vein; PVI, pulmonary vein isolation; TIA, transient ischaemic attack.

What’s new?

- In this nationwide real-world pulsed-field ablation (PFA) registry, higher body mass index (BMI) was associated with more comorbidities, more persistent atrial fibrillation (AF), and more extensive ablation strategies.
- Severe obesity was not associated with worse 1-year rhythm outcomes after PFA, and no statistically significant increase in periprocedural complications was observed.
- These findings suggest that baseline BMI alone should not be used as a reason to defer AF ablation, while comprehensive risk-factor management remains essential.

## Introduction

Atrial fibrillation (AF) is associated with morbidity, impaired quality of life, and repeated hospitalizations. In recent years, catheter ablation has gained an increasingly important role in rhythm-control strategies, and the latest European guidelines place it as a central therapy for symptomatic patients and, in selected cases, at earlier stages of the disease course.^1^ At the same time, these guidelines emphasize comprehensive management of AF beyond the procedure itself, with particular attention to comorbidities and risk-factor modification, especially weight reduction as part of an upstream therapy approach.^2^

Obesity is a key determinant of AF substrate. It is associated with incident AF, atrial remodelling, progression towards persistent forms, and a higher burden of cardiometabolic comorbidities. However, while the role of obesity in the natural history of AF is well established, the prognostic significance of baseline body weight at the time of ablation remains less clear. In some settings, obesity may influence referral patterns or perceived candidacy for ablation. It is uncertain whether obesity is associated with poorer rhythm outcomes after ablation, or with a higher risk of periprocedural complications, independently of the clinical profile that usually accompanies excess body weight.

This question is particularly relevant in the era of pulsed-field ablation (PFA), which has rapidly expanded into routine practice. The FRANCE-PFA registry demonstrated the feasibility of nationwide PFA implementation. Data specifically addressing outcomes according to baseline body mass index (BMI) remain limited. We therefore assessed the association between baseline BMI and 1-year outcomes after first-time PFA for AF in the FRANCE-PFA registry.

## Methods

### Study design and population

This study was based on the FRANCE-PFA registry, a prospective nationwide registry (NCT06497933) including patients undergoing first-time AF ablation using the pentaspline PFA catheter (FARAPULSE, Boston Scientific®) in France since the introduction of this technology from March 2021 to February 2024.^3^ All French centres using this technology, including academic, non-academic, public, and private institutions, participated in the registry. Patients undergoing redo AF ablation procedures were not included in the present study. Procedural data were prospectively collected at the patient level. All consecutive patients were included. The registry was conducted in accordance with the Declaration of Helsinki. Institutional review board approval was obtained at each participating institution. All patients provided informed consent, and data were anonymized before analysis.

For the present analysis, we included patients with available baseline BMI. Patients with missing BMI were excluded from BMI-based analyses.

### Procedures and data collection

Ablation procedures followed contemporary PFA practice, consistent with current expert consensus and state-of-the-art procedural principles, and have been described in detail in the original FRANCE-PFA report.^3–5^ All procedures were performed using the pentaspline PFA system (FARAPULSE, Boston Scientific). Beyond pulmonary vein isolation (PVI), additional lesion sets were performed at the operator’s discretion. When posterior wall ablation was performed, it consisted of overlapping applications extending from the pulmonary vein antra across the left atrial posterior wall, resulting in posterior wall ablation rather than line-based isolation. Roof and mitral isthmus lesion sets were similarly delivered at the operator’s discretion. The registry recorded the intended anatomical lesion set rather than the detailed spatial distribution of individual overlapping applications. Procedures were otherwise performed according to routine practice at participating centres. The registry prospectively collected baseline clinical characteristics, AF type, procedural characteristics, acute procedural data, and adverse events through an online database. In the original registry, centres were asked to confirm and detail complications, which were then reviewed and adjudicated by an independent committee.

For the present study, variables of interest included age, sex, sleep apnoea, hypertension, diabetes mellitus, prior stroke/transient ischaemic attack, vascular disease, structural heart disease, AF type, procedural lesion set, and periprocedural complications. Procedural strategy was characterized using PVI-only vs. PVI + additional lesion sets, including roof line, posterior wall, and mitral isthmus ablation when applicable.

### Body mass index definition

Baseline BMI was analysed using two complementary approaches. First, patients were described according to World Health Organization BMI categories: <25, 25 to <30, 30 to <35, and ≥35 kg/m^2^. Secondly, the main exposure for the primary effectiveness analysis was severe obesity, defined as BMI ≥ 35 kg/m^2^. Body mass index was also modelled as a continuous variable, scaled per 5 kg/m^2^ increase. Sensitivity analyses were performed for BMI extremes using BMI < 20 kg/m^2^ and BMI ≥ 40 kg/m^2^. The lower cut-off does not define underweight according to WHO criteria but was used to explore whether a low-BMI phenotype modified outcomes. Conversely, BMI ≥ 40 kg/m^2^ corresponds to Class III obesity.

### Study outcomes

The primary effectiveness outcome was time to first documented atrial arrhythmia recurrence within 12 months after the index procedure. Recurrence was defined as documented AF, atrial flutter, or atrial tachycardia, generally lasting >30 s, according to standard clinical practice at each participating centre. Follow-up was not protocol mandated and reflected routine practice, including outpatient visits, telephone follow-up, review of symptoms, standard ECGs, ambulatory Holter ECG monitoring, or other rhythm documentation when clinically indicated. Patients were followed from the index procedure until the first documented atrial arrhythmia recurrence, last available follow-up, or 12 months, whichever occurred first.

### Statistical analysis

Continuous variables were expressed as mean ± SD or median [interquartile range], as appropriate. For descriptive comparisons across BMI categories, categorical variables were compared using the χ^2^ test or Fisher’s exact test, and continuous variables using analysis of variance or the Kruskal–Wallis test, as appropriate. Percentages for categorical variables were calculated using non-missing denominators within each BMI category.

The primary effectiveness analysis was based on Kaplan–Meier estimates of recurrence-free survival according to severe obesity status (BMI ≥ 35 kg/m^2^), with comparison by log-rank test. For time-to-recurrence analyses, the primary approach was a Cox proportional hazards model, which estimates cause-specific hazards for arrhythmia recurrence. The proportional hazards assumption was assessed using Schoenfeld residuals and corresponding global and variable-specific tests. Two main Cox modelling strategies were used: a categorical BMI model using BMI ≥ 35 kg/m^2^ and a continuous BMI model using BMI per 5 kg/m^2^ increase. Patients without documented recurrence were censored at the date of the last available follow-up, and patients followed beyond 12 months were administratively censored at 12 months. Events occurring after 12 months, if any, were not counted in the primary 1-year analysis. Univariable and multivariable Cox models were fitted in the same complete-case population to ensure comparability of estimates.

The main multivariable models were adjusted for age, sex, sleep apnoea, hypertension, diabetes mellitus, prior stroke/transient ischaemic attack, vascular disease, structural heart disease, and AF type. Separate sensitivity models additionally adjusted the main multivariable model for PVI-only strategy or baseline amiodarone use. To explore potential non-linearity in the association between BMI and recurrence, BMI was modelled using restricted cubic splines with knots placed at the 5th, 25th, 75th, and 95th percentiles of the BMI distribution. Effect modification by ablation strategy was explored by adding a multiplicative interaction term between severe obesity (BMI ≥ 35 kg/m^2^) and PVI-only strategy in the multivariable Cox model. The interaction was assessed using a Wald test. Periprocedural complications were compared descriptively across BMI categories. Periprocedural safety was primarily assessed descriptively by comparing overall and major complication rates across BMI categories using χ^2^ tests or Fisher’s exact tests when expected cell counts were small. Sensitivity analyses were performed using the conventional obesity threshold of BMI ≥ 30 kg/m^2^. To assess the robustness of the findings to potential centre-level effects, additional Cox models were fitted using robust standard errors clustered by centre and Cox models stratified by centre, using the same covariates as the multivariable model additionally adjusted for PVI-only strategy. Because individual complications were rare, no separate multivariable models were planned for each complication subtype. If event counts allowed, an exploratory logistic regression model was used for overall or major complications. Because BMI was the main exposure and was missing in a subset of patients, the primary analysis was performed as a complete-case analysis. As a sensitivity analysis, missing BMI values were handled using multiple imputation by chained equations under a missing-at-random assumption. Body mass index was imputed as a continuous variable using predictive mean matching with five nearest neighbours. The imputation model included baseline covariates, PVI-only strategy, recurrence status, follow-up time, and the Nelson–Aalen cumulative hazard estimate. All statistical tests were two sided, and a *P* value of <0.05 was considered statistically significant. Analyses were performed using Stata software, version 15.1 (StataCorp, College Station, TX, USA).

## Results

### Study population

A total of 5223 patients underwent PFA for AF during the study period. Baseline BMI was available in 4573 patients (87.6%), and the mean BMI was 27.6 ± 5.1 kg/m^2^. According to BMI categories, 1511 patients (33.0%) had BMI < 25 kg/m^2^, 1775 (38.8%) had BMI of 25 to <30 kg/m^2^, 882 (19.3%) had BMI of 30 to <35 kg/m^2^, and 405 (8.9%) had BMI ≥ 35 kg/m^2^. Follow-up information for arrhythmia recurrence was available in 4262 patients with baseline BMI.

### Baseline characteristics according to body mass index category

Higher BMI categories were associated with a higher burden of comorbidities, including sleep apnoea, which increased from 4.8% in patients with BMI < 25 kg/m^2^ to 35.6% in those with BMI ≥ 35 kg/m^2^ (*P* < 0.001), arterial hypertension (29.0–57.3%, *P* < 0.001), and diabetes mellitus (5.6–25.4%, *P* < 0.001) (*Table 1*). Higher BMI was also associated with moderate to severe atrial dilatation (48.0% vs. 72.4%, *P* < 0.001). The prevalence of non-paroxysmal AF increased across BMI categories (from 34.5 to 58.4%, *P* < 0.001). Baseline characteristics of patients with missing BMI are described in Supplementary material online, *Table S1*.

**Table 1:** Baseline characteristics according to BMI category

Characteristic	Overall (*N* = 4573)	BMI < 25 kg/m^2^ (*n* = 1511)	BMI 25 to <30 kg/m^2^ (*n* = 1775)	BMI 30 to <35 kg/m^2^ (*n* = 882)	BMI ≥ 35 kg/m^2^ (*n* = 405)	*P* value
**Age, years**	65.1 ± 11.2	65.8 ± 11.9	65.5 ± 10.8	64.2 ± 10.5	62.7 ± 10.7	<0.001
**Female sex**	1420 (31.2%)	551 (36.5%)	432 (24.5%)	262 (29.8%)	175 (43.3%)	<0.001
**BMI, kg/m^2^**	27.6 ± 5.1	22.6 ± 1.8	27.2 ± 1.4	32.0 ± 1.4	38.8 ± 3.4	<0.001
**Hypertension**	1807 (39.5%)	438 (29.0%)	708 (39.9%)	429 (48.6%)	232 (57.3%)	<0.001
**Diabetes mellitus**	502 (11.0%)	85 (5.6%)	152 (8.6%)	162 (18.4%)	103 (25.4%)	<0.001
**Sleep apn**o**ea**	603 (13.2%)	73 (4.8%)	204 (11.5%)	182 (20.6%)	144 (35.6%)	<0.001
**Prior stroke/TIA**	272 (5.9%)	93 (6.2%)	106 (6.0%)	57 (6.5%)	16 (4.0%)	0.331
**Vascular disease**	294 (6.4%)	87 (5.8%)	120 (6.8%)	57 (6.5%)	30 (7.4%)	0.551
**CHA2DS2-VASc score**	1.9 ± 1.5	1.9 ± 1.4	1.8 ± 1.4	2.0 ± 1.5	2.2 ± 1.4	<0.001
**Structural heart disease**	1273 (27.8%)	379 (25.1%)	488 (27.5%)	269 (30.5%)	137 (33.8%)	0.001
**LVEF > 50%**	2788 (77.9%)	927 (81.0%)	1102 (78.4%)	531 (74.7%)	228 (72.4%)	<0.001
**Moderate/severe atrial dilation**	1659 (57.0%)	465 (48.0%)	652 (56.5%)	366 (66.9%)	176 (72.4%)	<0.001
**Paroxysmal AF**	2388 (54.5%)	950 (65.5%)	909 (53.4%)	367 (43.6%)	162 (41.6%)	<0.001
**Persistent AF**	1700 (38.8%)	421 (29.0%)	691 (40.6%)	401 (47.6%)	187 (48.1%)	<0.001
**Long-standing persistent AF**	294 (6.7%)	79 (5.4%)	101 (5.9%)	74 (8.8%)	40 (10.3%)	<0.001
**Any antiarrhythmic drug**	4335 (94.8%)	1414 (93.6%)	1686 (95.0%)	846 (95.9%)	389 (96.0%)	0.042
**Class I antiarrhythmic drug**	932 (20.4%)	363 (24.0%)	367 (20.7%)	144 (16.3%)	58 (14.3%)	<0.001
**Amiodarone**	1555 (34.0%)	449 (29.7%)	580 (32.7%)	357 (40.5%)	169 (41.7%)	<0.001

AF, atrial fibrillation; BMI, body mass index; LVEF, left ventricular ejection fraction; TIA, transient ischaemic attack.

### Procedural characteristics

When comparing the lowest and highest BMI categories, the proportion of PVI-only procedures was lower in patients with BMI ≥ 35 kg/m^2^ than in those with BMI < 25 kg/m^2^ (53.1% vs. 65.1%; overall *P* < 0.001 across BMI categories) (*Table 2*). Conversely, any additional lesion set was more frequently performed in patients with BMI ≥ 35 kg/m^2^ than in those with BMI < 25 kg/m^2^ (44.4% vs. 31.5%; overall *P* < 0.001 across BMI categories). The number of extra-pulmonary vein applications also increased across BMI categories, from 6.9 ± 13.0 in patients with BMI < 25 kg/m^2^ to 12.1 ± 19.9 in those with BMI ≥ 35 kg/m^2^ (overall *P* < 0.001). Similarly, the total number of PFA applications increased from 49.6 ± 20.2 to 56.2 ± 27.0 across the same BMI categories (overall *P* < 0.001). Among additional lesion sets, roof lines increased from 23.3% in patients with BMI < 25 kg/m^2^ to 30.9% in those with BMI ≥ 35 kg/m^2^ (*P* = 0.011), and posterior wall ablation increased from 16.7 to 23.7% across the same BMI categories (*P* = 0.006). Fluoroscopy dose-area product increased progressively across BMI categories (*P* < 0.001). Procedure time, fluoroscopy time, and the number of venous applications did not differ significantly across groups.

**Table 2:** Procedural characteristics according to BMI category

Procedural characteristics	Overall (*N* = 4573)	BMI < 25 kg/m^2^ (*n* = 1511)	BMI 25 to <30 kg/m^2^ (*n* = 1775)	BMI 30 to <35 kg/m^2^ (*n* = 882)	BMI ≥ 35 kg/m^2^ (*n* = 405)	*P* value
**Atropine use**	1748 (38.2%)	573 (37.9%)	693 (39.0%)	337 (38.2%)	145 (35.8%)	0.667
**Isoproterenol use**	1 (0.0%)	1 (0.1%)	0 (0.0%)	0 (0.0%)	0 (0.0%)	0.567
**Flecainide use**	6 (0.1%)	3 (0.2%)	1 (0.1%)	1 (0.1%)	1 (0.2%)	0.633
**PVI-only strategy**	2817 (61.6%)	984 (65.1%)	1111 (62.6%)	507 (57.5%)	215 (53.1%)	<0.001
**Non-paroxysmal AF with PVI-only strategy**	879 (19.2%)	214 (14.2%)	354 (19.9%)	223 (25.3%)	88 (21.7%)	<0.001
**Any additional lesion set**	1533 (35.2%)	452 (31.5%)	590 (34.7%)	319 (38.6%)	172 (44.4%)	<0.001
**Roof line**	1165 (25.5%)	352 (23.3%)	450 (25.4%)	238 (27.0%)	125 (30.9%)	0.011
**Posterior wall ablation**	864 (18.9%)	252 (16.7%)	335 (18.9%)	181 (20.5%)	96 (23.7%)	0.006
**Mitral isthmus line**	309 (6.8%)	107 (7.1%)	112 (6.3%)	69 (7.8%)	21 (5.2%)	0.262
**Procedure time, min**	54.7 ± 26.6	55.5 ± 26.7	53.8 ± 24.6	55.1 ± 30.8	55.0 ± 24.4	0.346
**Fluoroscopy time, min**	13.4 [9.4–19.3]	13.5 [9.5–19.1]	13.0 [9.0–19.8]	14.0 [9.6–19.0]	14.0 [9.8–19.4]	0.462
**Fluoroscopy dose-area product, Gy·cm^2^**	4.7 [2.3–9.8]	3.6 [1.7–6.7]	4.7 [2.4–9.7]	5.9 [2.9–14.1]	8.6 [4.0–18.7]	<0.001
**Number of venous applications**	42.8 ± 12.6	42.7 ± 12.2	42.6 ± 12.8	42.6 ± 12.7	44.2 ± 13.3	0.143
**Number of extra-pulmonary vein applications**	8.4 ± 15.5	6.9 ± 13.0	8.2 ± 15.8	9.4 ± 16.2	12.1 ± 19.9	<0.001
**Total number of PFA applications**	51.1 ± 22.7	49.6 ± 20.2	50.8 ± 23.3	51.9 ± 23.2	56.2 ± 27.0	<0.001

AF, atrial fibrillation; BMI, body mass index; PFA, pulsed-field ablation; PVI, pulmonary vein isolation.

### Periprocedural complications

Periprocedural complications occurred in 44 of 4573 procedures (1.0%). The rate of any complication did not differ significantly across BMI categories. Cardiac tamponade requiring drainage occurred in 18 patients with available BMI data (*Table 3*). Of these, 12 were managed percutaneously and six surgically. Percutaneous vs. surgical management was observed in two patients vs. one patient with BMI < 25 kg/m^2^, six vs. two with BMI of 25 to <30 kg/m^2^, three vs. one with BMI of 30 to <35 kg/m^2^, and one vs. two with BMI ≥ 35 kg/m^2^, respectively. One patient with BMI ≥ 35 kg/m^2^ died following surgical drainage of the tamponade. The second periprocedural death occurred in a patient with BMI < 25 kg/m^2^ and was due to refractory heart failure on the day after the procedure. In multivariable logistic regression adjusted for age, sex, sleep apnoea, structural heart disease, and AF type, severe obesity was not significantly associated with periprocedural complications (OR, 1.73; 95% CI, 0.73–4.11; *P* = 0.213). Severe obesity was not significantly associated with major complications after multivariable adjustment (OR, 1.46; 95% CI, 0.53–4.02; *P* = 0.468).

**Table 3:** Periprocedural complications according to BMI category

Complication	Overall (*N* = 4573)	BMI < 25 kg/m^2^ (*n* = 1511)	BMI 25 to <30 kg/m^2^ (*n* = 1775)	BMI 30 to <35 kg/m^2^ (*n* = 882)	BMI ≥ 35 kg/m^2^ (*n* = 405)	*P* value
Any adjudicated complication	44 (1.0%)	10 (0.7%)	17 (1.0%)	10 (1.1%)	7 (1.7%)	0.219
Major safety composite^a^	26 (0.6%)	7 (0.5%)	9 (0.5%)	6 (0.7%)	4 (1.0%)	0.539
Cardiac tamponade requiring drainage	18 (0.4%)	3 (0.2%)	8 (0.5%)	4 (0.5%)	3 (0.7%)	0.292
Vascular complication requiring intervention	8 (0.2%)	1 (0.1%)	5 (0.3%)	1 (0.1%)	1 (0.2%)	0.408
Stroke/TIA	7 (0.2%)	3 (0.2%)	1 (0.1%)	2 (0.2%)	1 (0.2%)	0.388
Death	2 (<0.1%)	1 (0.1%)	0 (0.0%)	0 (0.0%)	1 (0.2%)	0.141
Ventricular arrhythmia	2 (<0.1%)	0 (0.0%)	1 (0.1%)	0 (0.0%)	1 (0.2%)	0.201
Coronary spasm	4 (0.1%)	0 (0.0%)	1 (0.1%)	2 (0.2%)	1 (0.2%)	0.119
Haemoptysis	2 (<0.1%)	0 (0.0%)	1 (0.1%)	1 (0.1%)	0 (0.0%)	0.592

BMI, body mass index; TIA, transient ischaemic attack.

^a^Major safety composite was defined as cardiac tamponade requiring drainage, stroke/TIA, or death.

### Primary effectiveness analysis

Follow-up information for arrhythmia recurrence was available in 4262 patients with baseline BMI. Among these, 199 had missing data for at least one covariate included in the multivariable model, leaving 4063 patients in the complete-case cohort used for the primary effectiveness analysis. In this cohort, 699 experienced arrhythmia recurrence. Recurrence-free survival did not differ according to severe obesity status (log-rank *P* = 0.925). At 12 months, estimated recurrence rates were driven by AF type rather than BMI: 13.8% in patients with paroxysmal AF and BMI < 35 kg/m^2^, 9.1% in those with paroxysmal AF and BMI ≥ 35 kg/m^2^, 22.9% in those with non-paroxysmal AF and BMI < 35 kg/m^2^, and 23.3% in those with non-paroxysmal AF and BMI ≥ 35 kg/m^2^ (*Figure 1*).

**Figure 1:**
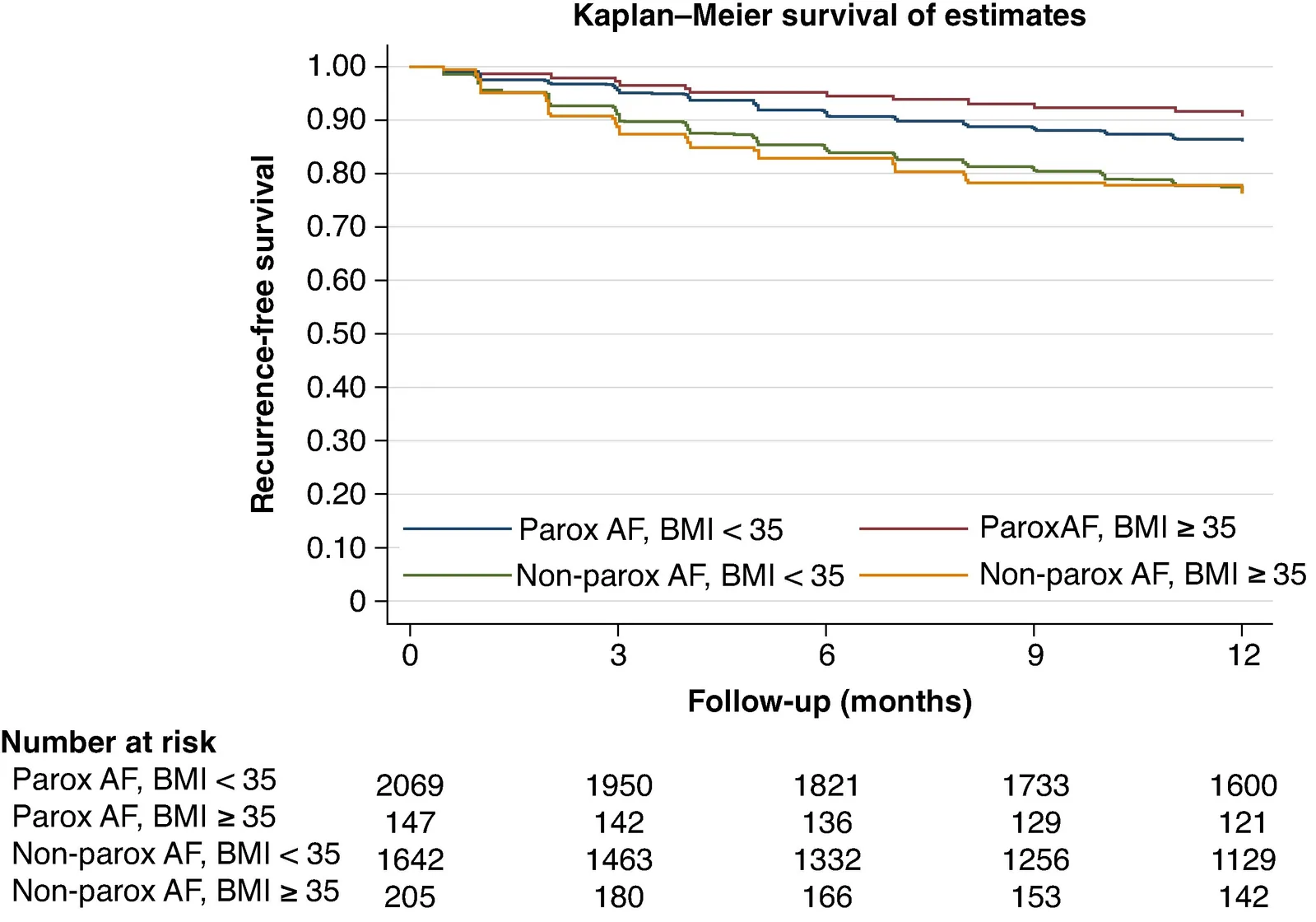
Recurrence-free survival according to AF type and baseline BMI. Kaplan–Meier curves are shown for paroxysmal AF with BMI < 35 kg/m^2^, paroxysmal AF with BMI ≥ 35 kg/m^2^, non-paroxysmal AF with BMI < 35 kg/m^2^, and non-paroxysmal AF with BMI ≥ 35 kg/m^2^. Follow-up was censored at 12 months. AF, atrial fibrillation; BMI, body mass index.

A BMI of ≥35 kg/m^2^ was not associated with arrhythmia recurrence in univariate analysis (HR, 0.99; 95% CI, 0.76–1.29; *P* = 0.925), after multivariable adjustment (HR, 0.86; 95% CI, 0.65–1.14; *P* = 0.299), or in separate sensitivity models additionally adjusted for PVI-only strategy (HR, 0.86; 95% CI, 0.65–1.14; *P* = 0.292) or baseline amiodarone use (HR, 0.87; 95% CI, 0.66–1.15; *P* = 0.337; Supplementary material online, *Table S4*). When BMI was analysed as a continuous variable, no significant association was observed in univariable analysis (HR per 5 kg/m^2^ increase, 0.99; 95% CI, 0.92–1.07; *P* = 0.785), multivariable analysis (HR, 0.93; 95% CI, 0.86–1.01; *P* = 0.076), or in separate sensitivity models additionally adjusted for PVI-only strategy (HR, 0.93; 95% CI, 0.86–1.01; *P* = 0.075) or baseline amiodarone use (HR per 5 kg/m^2^ increase, 0.94; 95% CI, 0.86–1.01; *P* = 0.104; Supplementary material online, *Table S4*) (*Table 4*). Restricted cubic spline analysis did not show a non-linear association between BMI and recurrence risk (*P* for non-linearity = 0.629). No significant interaction was observed between severe obesity and AF type, sleep apnoea status, or PVI-only strategy for the risk of recurrence (*P* for interaction = 0.158, 0.327, and 0.165, respectively; Supplementary material online, *Table S3*). Sensitivity analyses using multiple imputation for missing BMI values yielded similar results: neither severe obesity (HR, 0.86; 95% CI, 0.66–1.13; *P* = 0.283) nor BMI analysed continuously per 5 kg/m^2^ increase (HR, 0.93; 95% CI, 0.86–1.01; *P* = 0.082) was associated with 1-year arrhythmia recurrence (see Supplementary material online, *Table S4*). Sensitivity analyses yielded consistent results when obesity was defined using the conventional BMI ≥ 30 kg/m^2^ threshold and when accounting for centre effects (see Supplementary material online, *Table S5*).

**Table 4:** Association between baseline BMI and arrhythmia recurrence after PFA

Exposure	Model	HR	95% CI	*P* value
**Severe obesity (BMI ≥ 35 kg/m^2^)**	Univariable	0.99	0.76–1.29	0.925
**Severe obesity (BMI ≥ 35 kg/m^2^)**	Multivariable*	0.86	0.65–1.14	0.299
**Severe obesity (BMI ≥ 35 kg/m^2^)**	Multivariable + PVI-only	0.86	0.65–1.14	0.292
**BMI per 5 kg/m^2^ increase**	Univariable	0.99	0.92–1.07	0.785
**BMI per 5 kg/m^2^ increase**	Multivariable*	0.93	0.86–1.01	0.076
**BMI per 5 kg/m^2^ increase**	Multivariable + PVI-only	0.93	0.86–1.01	0.075
**BMI non-linearity (restricted cubic spline)**	Adjusted model*	–	–	0.629

BMI, body mass index; CI, confidence interval; HR, hazard ratio; PFA, pulsed-field ablation; PVI, pulmonary vein isolation.

*Adjusted for age, sex, sleep apnoea, hypertension, diabetes mellitus, prior stroke/transient ischaemic attack, vascular disease, structural heart disease, and AF type.

### Extreme body mass index

Sensitivity analyses for BMI extremes are shown in Supplementary material online, *Table S2*. Among 134 patients with BMI < 20 kg/m^2^, 19 (14.2%) experienced arrhythmia recurrence; BMI < 20 kg/m^2^ was not associated with 1-year arrhythmia recurrence in adjusted analysis (HR, 0.79; 95% CI, 0.50–1.25; *P* = 0.308). Among 94 patients with BMI ≥ 40 kg/m^2^, 17 (18.1%) experienced arrhythmia recurrence; BMI ≥ 40 kg/m^2^ was also not associated with recurrence (HR, 0.90; 95% CI, 0.55–1.47; *P* = 0.676). These results were similar after additional adjustment for PVI-only strategy.

## Discussion

In this prospective nationwide cohort of patients undergoing first-time PFA for AF, higher BMI was associated with a more advanced clinical phenotype. Despite this less favourable baseline profile, severe obesity was not independently associated with 1-year arrhythmia recurrence after adjustment for clinical characteristics and procedural strategy. No statistically significant increase in periprocedural complications was observed, although safety analyses were limited by the low number of events. These findings were consistent across subgroup and extreme BMI analyses.

Our results challenge the assumption that obesity *per se* should be considered a marker of reduced ablation efficacy.^1^ Obesity is strongly associated with atrial remodelling, cardiometabolic burden, and AF progression.^6,7^ From a pathophysiological perspective, it would therefore be expected to adversely influence ablation outcomes. This expectation is supported by historical radiofrequency series and meta-analyses, which reported a progressive increase in post-ablation recurrence with increasing BMI, especially beyond 35–40 kg/m^28–10^. However, contemporary data suggest a more nuanced interpretation. In real-world thermal ablation cohorts, BMI is associated with a broader clinical phenotype, whereas its independent contribution to recurrence is less consistent. Tabaja *et al.*^11^ reported that catheter ablation remained safe across BMI categories and improved quality of life in all groups, although Class III obesity was associated with lower sinus rhythm maintenance success. Vermeer *et al.*^12^ similarly found no independent association between BMI and procedural complications, despite more repeat procedures and lower quality of life in obese patients. Conversely, cryoballoon data from the Korean national registry argue against a monotonic relationship, with increased recurrence predominantly among underweight patients.^13^

The present findings extend this view to PFA. The EU-PORIA registry suggested a potential association between higher BMI and recurrence after PFA, whereas in the FRANCE-PFA registry, severe obesity was not independently associated with 1-year recurrence after adjustment for AF type, comorbidities, structural heart disease, and procedural strategy.^14^ Body mass index at the time of ablation may therefore be an incomplete surrogate for the cumulative impact of obesity on the atrial substrate. It does not capture obesity duration, weight trajectory, visceral or epicardial adiposity, fitness, sleep apnoea control, or prior risk-factor modification. From a procedural standpoint, PFA does not rely on resistive or conductive heating and may be less susceptible to some mechanisms traditionally invoked to explain variability in thermal lesion formation. This does not establish superiority over thermal ablation, and current comparative evidence remains mixed; however, PFA has consistently been associated with procedural efficiency and, in some cases, better rhythm outcomes compared with cryoballoon ablation in obese populations.^15,16^

Our findings should therefore not be interpreted as evidence against the role of obesity in AF progression or against risk-factor management. Rather, they should be considered within the integrated AF-CARE framework of the 2024 ESC/EACTS guidelines,^2^ in which comorbidity and risk-factor management represents a central component of AF care. However, implementation remains variable in contemporary electrophysiology practice. In a recent EHRA survey, only 23.5% of patients with AF and obesity were estimated to be referred for formal dietary advice, while 37.7% of respondents reported using a BMI threshold when determining suitability for AF ablation.^17^ These observations highlight the persisting gap between recommended risk-factor management and its implementation in routine practice.

Intensive management, including structured risk-factor modification, bariatric surgery, and more recently glucagon-like peptide-1 (GLP-1) receptor agonist-based approaches, has been associated with lower AF burden or lower recurrence after ablation^7,18–20^. These interventions address a different question from the one examined here.^21^ They aim to modify the long-term cardiometabolic and atrial substrate, cornerstones of AF management,^1^ whereas our analysis assessed whether BMI at the time of PFA independently predicted procedural safety or 1-year rhythm outcome. These two approaches should be considered complementary rather than sequential or mutually exclusive.

This distinction has direct implications for patient selection and access to rhythm-control therapy. In patients with uncontrolled or unstable comorbidities, temporary deferral of an invasive rhythm-control strategy may be appropriate while risk factors are optimized. However, obesity itself represents a chronic condition and not an acute periprocedural instability. If baseline BMI alone is used as a reason to defer or deny ablation, patients with obesity may experience delayed access to rhythm-control therapy despite ongoing AF progression while remaining exposed to the long-term cardiometabolic consequences of obesity. In the present cohort, higher BMI identified patients with more comorbidities, more atrial dilatation, and more persistent AF, but did not identify patients with higher procedural complication rates or poorer 1-year rhythm outcome.

Patients with obesity are often perceived as being at higher procedural risk, owing to anticipated challenges in vascular access, sedation or anaesthesia, imaging quality, respiratory management, and post-procedural logistics. Such concerns may be clinically relevant in selected patients, but they should be assessed individually rather than inferred from BMI alone. In line with growing recognition of weight stigma and implicit bias in healthcare,^22^ therapeutic decisions should be guided by procedure-specific evidence rather than by assumptions extrapolated from the long-term risk profile associated with obesity.^23^

Patients with higher BMI underwent more extensive ablation strategies, with fewer PVI-only procedures and more frequent additional lesion sets. This observation likely reflects both the obesity-associated AF phenotype and operator adaptation to more advanced atrial disease. Importantly, adjustment for PVI-only strategy did not materially change the association between BMI and recurrence, and no significant interaction was observed between severe obesity and ablation strategy. Thus, the absence of an independent association between severe obesity and recurrence does not appear to be explained solely by differences in lesion-set selection.

Taken together, these findings support a nuanced clinical framework. Obesity remains a major therapeutic target in AF care, and weight reduction should remain part of comprehensive management. However, baseline BMI alone should not be equated with reduced PFA effectiveness or excess procedural risk. For many patients, rhythm-control intervention and upstream therapy may need to proceed in parallel: ablation to address the current electrical phenotype and risk-factor management to reduce long-term substrate progression and recurrence risk.

Future studies should also move beyond the conventional binary assessment of post-ablation recurrence. Time to first documented recurrence does not capture the amount or temporal pattern of recurrent AF and may incompletely characterize the effect of obesity on post-ablation rhythm outcomes. Quantification or estimation of AF burden, defined as the proportion of monitored time spent in AF over a specified monitoring period, may provide a more informative endpoint to investigate the relationship between cardiometabolic substrate and rhythm outcome after ablation.^24^

This study has several limitations. First, its observational design leaves it susceptible to residual confounding and selection bias. Patients with severe, particularly Class III obesity, were relatively under-represented, and those undergoing ablation may have constituted a selected subgroup considered suitable for the procedure by their treating teams. Accordingly, our findings should not be extrapolated to an unselected all-comer population with severe or Class III obesity, particularly patients with uncontrolled comorbidities or other factors that may lead operators to defer ablation. These findings therefore primarily apply to appropriately selected patients undergoing PFA in experienced AF ablation centres. Secondly, the analysis addressed baseline BMI only. No information was available on obesity duration, weight trajectory, post-ablation weight loss, GLP-1 receptor agonist use, bariatric surgery, structured risk-factor management, sleep apnoea treatment, fitness, or visceral and epicardial adiposity. Therefore, we cannot determine whether post-ablation obesity management or changes in cardiometabolic status influenced 1-year recurrence. Thirdly, BMI was missing in a subset of patients, who differed in several baseline and procedural characteristics, although crude recurrence rates were similar and multiple-imputation analyses yielded consistent results. Fourthly, procedural strategies differed across BMI categories, with more extensive lesion sets and a higher number of PFA applications in patients with higher BMI. Although adjustment for PVI-only ablation did not materially alter the findings, these differences may reflect both a more advanced AF substrate and operator perception or adaptation to obesity-associated procedural complexity. Fifthly, follow-up was performed according to routine practice at each participating centre rather than according to a standardized rhythm-monitoring protocol. Recurrence detection may therefore have varied across centres and according to clinical presentation, and asymptomatic recurrences may have been underestimated. Importantly, because symptom perception and comorbidity burden, including sleep apnoea, may differ according to obesity status, differential ascertainment of recurrence across BMI groups cannot be excluded. Comparisons with registries using more intensive surveillance should be made cautiously. Sixthly, the findings apply to 1-year outcomes only, and longer follow-up will be needed to determine whether BMI influences later arrhythmia recurrence or repeat ablation.

## Conclusions

In the nationwide FRANCE-PFA registry, higher BMI was associated with a more advanced cardiometabolic and AF phenotype, but baseline BMI did not independently predict periprocedural complications or 1-year arrhythmia recurrence after PFA. In appropriately selected patients, severe obesity alone should not be used as a reason to defer AF ablation, while comprehensive risk-factor management should remain an integral component of long-term AF care.

## Supplementary Material

euag288_Supplementary_Data

## Data Availability

The data underlying this article cannot be shared publicly due to ethical and regulatory restrictions related to patient-level registry data. De-identified data may be made available by the corresponding author upon reasonable request and subject to approval by the FRANCE-PFA registry steering committee and applicable regulatory requirements.
