# Supplementary material for: Impact of obesity on procedural safety and 1-year outcomes after pulsed-field ablation for atrial fibrillation
Source: Europace. 2026 Oct 29;28(10):euag288. doi: 10.1093/europace/euag288 (PMC13717914; doi:10.1093/europace/euag288)
Supplement: Supplementary file 1 [file euag288_supplementary_data.docx]

| **Characteristic** | **BMI available (n=4573)** | **BMI missing (n=650)** | **P value** |
| --- | --- | --- | --- |
| Female sex | 1420 (31.2%) | 179 (27.6%) | 0.068 |
| Sleep apnea | 603 (13.2%) | 77 (11.8%) | 0.342 |
| Hypertension | 1807 (39.5%) | 196 (30.2%) | <0.001 |
| Diabetes mellitus | 502 (11.0%) | 49 (7.5%) | 0.008 |
| Prior stroke/TIA | 272 (5.9%) | 30 (4.6%) | 0.173 |
| Vascular disease | 294 (6.4%) | 27 (4.2%) | 0.024 |
| Structural heart disease | 1273 (27.8%) | 129 (19.8%) | <0.001 |
| Paroxysmal AF | 2388 (54.5%) | 375 (62.4%) | <0.001 |
| Persistent AF | 1700 (38.8%) | 200 (33.3%) | 0.009 |
| Long-standing persistent AF | 294 (6.7%) | 26 (4.3%) | 0.025 |
| PVI-only strategy | 2817 (61.6%) | 490 (75.3%) | <0.001 |
| Arrhythmia recurrence during follow-up | 727/4262 (17.1%) | 104/592 (17.6%) | 0.758 |
| Age, years | 65.1 ± 11.2 | 64.2 ± 11.2 | 0.068 |

Values are n (%) or mean ± SD. AF indicates atrial fibrillation; BMI, body mass index; PVI, pulmonary vein isolation; TIA, transient ischemic attack.

Supplementary Table 1. Characteristics of patients with available versus missing BMI. Values are n (%) or mean ± SD. AF indicates atrial fibrillation; BMI, body mass index; PVI, pulmonary vein isolation; TIA, transient ischemic attack.

| **Exposure** | **Patients, n** | **12-month recurrence, n (%)** | **Model** | **HR** | **95% CI** | **P value** |
| --- | --- | --- | --- | --- | --- | --- |
| BMI <20 kg/m² | 134 | 19 (14.2%) | Univariable | 0.79 | 0.50–1.25 | 0.323 |
| BMI <20 kg/m² | 134 | 19 (14.2%) | Multivariable | 0.79 | 0.50–1.25 | 0.308 |
| BMI <20 kg/m² | 134 | 19 (14.2%) | Multivariable + PVI-only | 0.79 | 0.49–1.25 | 0.306 |
| BMI ≥40 kg/m² | 94 | 17 (18.1%) | Univariable | 1.04 | 0.64–1.68 | 0.875 |
| BMI ≥40 kg/m² | 94 | 17 (18.1%) | Multivariable | 0.90 | 0.55–1.47 | 0.676 |
| BMI ≥40 kg/m² | 94 | 17 (18.1%) | Multivariable + PVI-only | 0.90 | 0.55–1.47 | 0.668 |

Supplementary Table 2. Sensitivity analyses according to extreme BMI categories. Values are hazard ratios for arrhythmia recurrence. The multivariable model was adjusted for age, sex, sleep apnea, hypertension, diabetes mellitus, prior stroke/transient ischemic attack, vascular disease, structural heart disease, and AF type. The sensitivity model additionally included PVI-only strategy. AF indicates atrial fibrillation; BMI, body mass index; CI, confidence interval; HR, hazard ratio; PVI, pulmonary vein isolation.

| **Interaction tested** | **Degrees of freedom** | **Chi-square** | **P for interaction** |
| --- | --- | --- | --- |
| Severe obesity × AF type | 2 | 3.69 | 0.158 |
| Severe obesity × sleep apnea | 1 | 0.96 | 0.327 |
| Severe obesity × PVI-only strategy | 1 | 1.92 | 0.165 |

Supplementary Table 3. Interaction analyses for one-year arrhythmia recurrence. Interaction analyses were performed using multiplicative interaction terms in adjusted Cox models. Severe obesity was defined as BMI ≥35 kg/m². Models were adjusted for age, sex, sleep apnea, hypertension, diabetes mellitus, prior stroke/transient ischemic attack, vascular disease, structural heart disease, and AF type, as appropriate. AF indicates atrial fibrillation; BMI, body mass index; PVI, pulmonary vein isolation.

| **Exposure** | **Analysis** | **Patients, n** | **Events, n** | **HR** | **95% CI** | **P value** |
| --- | --- | --- | --- | --- | --- | --- |
| Severe obesity, BMI ≥35 kg/m² | Complete-case adjusted model | 4,063 | 699 | 0.86 | 0.65–1.14 | 0.299 |
| Severe obesity, BMI ≥35 kg/m² | Adjusted model + baseline amiodarone | 4,063 | 699 | 0.87 | 0.66–1.15 | 0.337 |
| Severe obesity, BMI ≥35 kg/m² | Multiple-imputation adjusted model | 4,594 | 792 | 0.86 | 0.66–1.13 | 0.283 |
| BMI per 5 kg/m² increase | Complete-case adjusted model | 4,063 | 699 | 0.93 | 0.86–1.01 | 0.076 |
| BMI per 5 kg/m² increase | Adjusted model + baseline amiodarone | 4,063 | 699 | 0.94 | 0.86–1.01 | 0.104 |
| BMI per 5 kg/m² increase | Multiple-imputation adjusted model | 4,594 | 792 | 0.93 | 0.86–1.01 | 0.082 |

**Supplementary Table 4. Complete-case, amiodarone-adjusted, and multiple-imputation analyses.** Multiple imputation was performed using chained equations with predictive mean matching and 5 nearest neighbors for baseline BMI. The imputation model included baseline covariates, PVI-only strategy, recurrence status, follow-up time, and the Nelson–Aalen cumulative hazard estimate. Adjusted models included age, sex, sleep apnea, hypertension, diabetes mellitus, prior stroke/transient ischemic attack, vascular disease, structural heart disease, and AF type. The amiodarone sensitivity models additionally included baseline amiodarone use. AF indicates atrial fibrillation; BMI, body mass index; CI, confidence interval; HR, hazard ratio; PVI, pulmonary vein isolation; TIA, transient ischemic attack.

| **Exposure** | **Analysis** | **Patients, n** | **Events, n** | **HR** | **95% CI** | **P value** |
| --- | --- | --- | --- | --- | --- | --- |
| BMI ≥30 kg/m² | Univariable Cox model | 4,063 | 699 | 1.02 | 0.87–1.21 | 0.773 |
| BMI ≥30 kg/m² | Multivariable Cox model | 4,063 | 699 | 0.91 | 0.76–1.08 | 0.271 |
| BMI ≥30 kg/m² | Multivariable Cox model + PVI-only strategy | 4,063 | 699 | 0.91 | 0.76–1.08 | 0.269 |
| BMI ≥30 kg/m² | Multivariable Cox model + PVI-only strategy, with robust standard errors clustered by center | 4,063 | 699 | 0.91 | 0.75–1.10 | 0.316 |
| BMI ≥30 kg/m² | Cox model stratified by center, adjusted for clinical covariates and PVI-only strategy | 4,063 | 699 | 0.90 | 0.75–1.08 | 0.244 |

Supplementary Table 5. Sensitivity analyses using the conventional BMI ≥30 kg/m² obesity threshold and accounting for center effects. Values are hazard ratios for one-year atrial arrhythmia recurrence. BMI ≥30 kg/m² was compared with BMI <30 kg/m². The multivariable model was adjusted for age, sex, sleep apnea, hypertension, diabetes mellitus, prior stroke/transient ischemic attack, vascular disease, structural heart disease, and AF type. The sensitivity model additionally included PVI-only strategy. Center-sensitive analyses used the same covariates as the multivariable + PVI-only model, with either robust standard errors clustered by center or stratification by center. AF indicates atrial fibrillation; BMI, body mass index; CI, confidence interval; HR, hazard ratio; PVI, pulmonary vein isolation; TIA, transient ischemic attack.
